# Low Back Pain

**DOI:** 10.1155/2012/165479

**Published:** 2012-04-18

**Authors:** Seiji Ohtori, Yasuchika Aoki, Gen Inoue, Laura S. Stone, Giustino Varrassi

**Affiliations:** ^1^Department of Orthopaedic Surgery, Graduate School of Medicine, Chiba University, 1-8-1 Inohana, Chuo-ku, Chiba 260-8670, Japan; ^2^Department of Orthopaedic Surgery, Sakura Medical Center, Toho University, 654-1 Shimoshizu, Sakura, Chiba, 285-8741, Japan; ^3^Department of Orthopaedic Surgery, School of Medicine, Kitasato University, Kitasato 1-15-1, Sagamihara 252-0375, Japan; ^4^The Alan Edwards Centre for Research on Pain, Faculty of Dentistry, McGill University, 740 Dr. Penfield Avenue Suite 3200, Montreal, QC, Canada H3G 0G1; ^5^Universita degli Studi dell'Aquila, 67100 L'Aquila, Italy

Low back pain (LBP) is a common clinical problem and is of major socioeconomic importance. However, at the current time, there is little information about the pathogenesis of this disease. Furthermore, treatment option of LBP has not been fully explored. We focused on articles that will stimulate the continuing efforts to understand the pathology, epidemiology, diagnosis, and method of treatment of LBP.

We prepared 7 articles in this special issue as follows: “A novel image-guided, automatic high-intensity neurostimulation device for the treatment of nonspecific low back pain” by E. Schiff et al., “Evaluation of diagnosis techniques used for spinal injury related back pain” by E. Janssen et al., “Prevalence, spinal alignment, and mobility of lumbar spinal stenosis with or without chronic low back pain: a community-dwelling study” by Y. Kasukawa et al., “Assessment and treatment of abuse risk in opioid prescribing for chronic pain” by Serraillier et al., “Pain-related fear: a critical review of the related measures” by Verbunt et al., “The influence of pain distribution on walking velocity and horizontal ground reaction forces in patients with low back pain” by Etnyre et al.,” Stop using the modified work apgar to measure job satisfaction” by DeVellis et al.

We believe that these 7 articles help readers to understand the mechanism and therapy of chronic LBP.

*Seiji Ohtori* *Seiji Ohtori* *Yasuchika Aoki* *Yasuchika Aoki* *Gen Inoue* *Gen Inoue* *Laura S. Stone* *Laura S. Stone* *Giustino Varrassi* *Giustino Varrassi*

